# RNA-seq Splicing Profile of the *CDH1* Gene and Its Impact on the Clinical Pathogenicity Classification of *CDH1* Variants: A Description of Alternative and Pathogenic Splicing Patterns

**DOI:** 10.3390/cancers17203320

**Published:** 2025-10-14

**Authors:** Molka Sebai, Roseline Tang, Yahia Adnani, Alice Fievet, Odile Cabaret, Marie-Aude Robert de Rancher, Nathalie Auger, Yasmina Elaribi, Houweyda Jilani, Jean-Marc Limacher, Olivier Caron, Lamia Ben Jemaa, Etienne Rouleau

**Affiliations:** 1 Cancer Genetics Laboratory, Department of Medical Biology and Pathology, Gustave Roussy, 94805 Villejuif, France; 2 Department of Genetics, Mongi Slim Hospital, La Marsa 8030, Tunisia; yasmina.elaribi@fmt.utm.tn (Y.E.); lamia.benjemaa@fmt.utm.tn (L.B.J.); 3 Human Genetics Laboratory (LR99ES10), Faculty of Medicine of Tunis, University of Tunis el Manar, Tunis 2092, Tunisia; 4 Faculty of Medicine of Tunis, University of Tunis el Manar, Tunis 2092, Tunisia; 5 Laboratory of Fertility and Oncofertility LR16SP01, Aziza Othmana Hospital, Tunis 1008, Tunisia; 6 Maternal and Child Health Laboratory, LR22SP01-Mongi Slim Hospital, Tunis 8030, Tunisia; 7 Department of Medical Oncology, Hôpitaux Civils de Colmar, 68000 Colmar, France; jean-marc.limacher@ch-colmar.fr; 8 Department of Medical Oncology, Gustave Roussy, 94805 Villejuif, France; 9 INSERM U981, Gustave Roussy, 94805 Villejuif, France; 10 AMMICa UAR3655/US23, Gustave Roussy, 94805 Villejuif, France

**Keywords:** *CDH1* gene, alternative splicing, RNAseq, splicing variant classification

## Abstract

**Background/Objectives:** *CDH1* gene is widely studied, as pathogenic variants are involved in diffuse gastric cancers and lobular breast cancers. *CDH1* genotype contributes to the management of clinical practice recommendations for cancer prevention. We proposed a qualitative and quantitative description of *CDH1* alternative splicing profile on lymphoblastoid cell lines (LCLs). The aim of this description was to allow a comprehensive interpretation of the effect of variants of uncertain significance (VUS) on *CDH1* splicing. **Methods:** We studied, using RNAseq, the splicing profile of 22 LCLs (untreated and treated with puromycin) with no pathogenic variant on *CDH1* and evaluated the effect on *CDH1* splicing of four VUS. **Results:** We highlighted a total of eleven alternative splicing events including four junctions starting from intron 2, defining novel isoforms of *CDH1*. We also identified an isoform causing the skip of exon 11 and leading to a disruption of the reading frame with high levels of expression on negative *CDH1* control LCLs, confirmed by ddPCR. Splicing RNAseq results for *CDH1* VUS: c.1008+1G>A and c.1936+5G>A showed complex splicing patterns but allowed their classification as pathogenic. We studied *CDH1* VUS exon 4 to exon 11 duplication with RNA analysis combined with Bionano optical genome mapping. Depending on alternative splicing of proximal and distal exons 11 within the duplication, we identified four distinct transcripts, leading to truncated proteins, classifying the duplication as pathogenic. **Conclusions:** *CDH1* has a complex alternative splicing profile characterized by a dynamic splicing of intron 2 making *CDH1* a good candidate for a study using long-read RNAseq.

## Simple Summary

The *CDH1* gene encodes for E-cadherin, a key protein involved in cell adhesion. *CDH1* gene is frequently studied in the routine practice, to investigate a possible predisposition to breast and diffuse gastric cancers and to manage cancer prevention in the family. The point mutation and large rearrangement are studied. Herein, we presented a qualitative and quantitative description of alternative splicing events of *CDH1* gene using a short-read RNAseq approach. The aim of this work was to establish the *CDH1* alternative splicing profile as a basis for the interpretation of the possible clinical impact of *CDH1* splicing variants. We highlighted novel *CDH1* isoforms and presented an evaluation of known alternative *CDH1* isoforms. We validated our approach by classifying three pathogenic *CDH1* complex splicing variants. Our study findings suggest an interesting possible implication of *CDH1* intron 2 in CDH1 isoform regulation.

## 1. Introduction

The alternative RNA splicing is a biological mechanism of RNA variability that affects 90% of known genes [1]. A gene could have between tens to hundreds of alternative transcript isoforms with a qualitative and quantitative diversity of splicing events among the tissues [2]. Alternative splicing can give rise to isoforms that result in the production of truncated proteins. These potentially deleterious transcripts are typically targeted for degradation by the nonsense-mediated mRNA decay (NMD) pathway [3]. Several studies have demonstrated that the splicing profiles of cancer predisposition genes are altered in tumor cells compared to normal tissues [4].

Tumor cells ensure their survival using abnormal splicing by producing novel pathogenic splicing transcript isoforms or by increasing the level of expression of known alternative splicing isoforms, normally removed by the nonsense-mediated mRNA decay system [3]. Due to the diversity and the complexity of alternative RNA splicing, RNA sequencing (RNAseq) has shown a major potential for allowing a qualitative and quantitative evaluation of alternative and pathogenic splicing events [5]. The primary objective of this emerging approach is to enable the interpretation of variants of uncertain significance (VUS) that affect RNA splicing, particularly given their increased detection with the advent of next-generation DNA sequencing technologies [6,7]. Assessing their impact at the RNA level facilitates the evaluation and classification of the clinical pathogenicity of splicing-related VUS. However, the interpretation of RNA results could be challenging as it requires considering the gene expression in the studied tissue and the knowledge of the gene isoforms [8]. In routine practice, the interpretation of RNAseq data could face some major limits due to the lack of knowledge of alternative splicing profiles of the studied genes and the level of expression of their alternative isoforms [8]. Ideally, alternative splicing profiles must be established in the laboratory before starting a routine diagnosis RNA study activity. Actually, the description of physiological splicing profile of cancer predisposition genes have been mainly focused on hereditary breast and ovarian cancer (HBOC) genes [5,9,10].

In this work, we focused on the gene *CDH1* gene coding for E-cadherin. Pathogenic variants of the *CDH1* gene are involved in the predisposition to diffuse gastric cancer and lobular breast cancer [11]. In the tumor, we observe a loss of the E-cadherin protein due to the inactivation of *CDH1* gene by combining a first hit, mainly a truncating variant to a second hit causing the methylation of *CDH1* promotor (50%) or a loss of heterozygosity in favor of the pathogenic first hit (50%) [12,13,14].

The characterization of *CDH1* genotype status is important as it allows an adjustment of the medical follow-up for the mutated patients and avoids ineffective surveillance for the non-mutated relatives. The *CDH1* gene has been analyzed for many years in rare families where HDGC is suspected, but more recently, most of the routine HBOC panels have included this gene. The *CDH1* gene is therefore very frequently analyzed; increasing the chances to detect novel *CDH1* splicing variants which splicing effect must be documented by an RNA study. *CDH1* gene is characterized by a diverse splicing profile among the tissues [15] and some *CDH1* isoforms have been described in a dispersed manner in the literature [15,16].

The main purposes of this work were to provide a global qualitative and quantitative view of the alternative splicing isoforms of *CDH1* gene using a capture RNAseq diagnosis approach and to demonstrate the importance of this description for the classification of *CDH1* large rearrangements and potential *CDH1* splicing variants.

## 2. Materials and Methods

### 2.1. Selected Patients

#### 2.1.1. Description of CDH1 Alternative Splicing Patterns

We selected 22 lymphoblastoid cell lines (LCLs) of controls with no pathogenic variant on *CDH1* gene on DNA sequencing. The controls were carrying a variant with a predicted splicing effect on a cancer predisposition gene other than *CDH1*. They had also no personal nor family history suggesting an Hereditary Diffuse Gastric Cancer (HDGC) syndrome predisposition [17].

#### 2.1.2. Clinical and Family Data of Patients Carrying CDH1 Variants of Uncertain Significance with an Expected Effect on CDH1 Gene Splicing

Additionally, we studied the effect on *CDH1* gene splicing of four germline variants of uncertain significance (VUS) of *CDH1* gene from Gustave Roussy patients’ database: *CDH1* c.1008+1G>A; *CDH1* c.1936+5G>A; *CDH1* c.1566-10C>T and *CDH1* large duplication from exon 4 to exon 11. The splicing effect of the variants were studied by the in silico prediction scores: SPiP (https://github.com/raphaelleman/SPiP (accessed on 23 May 2025)) [18] and SpliceAI (https://spliceailookup.broadinstitute.org/ (accessed on 23 May 2025)) [19]. All the patients included in this study gave informed consent for genetic analysis.

We summarized the clinical data of the patients carrying these VUS in Table 1 below.

### 2.2. RNA Extraction

Total RNA was extracted from 22LCLs of negative controls and three LCLs of patients carrying *CDH1* VUS: *CDH1* c.1008+1G>A; *CDH1* c.1566-10C>T and *CDH1* large duplication from exon 4 to exon 11. LCLs were immortalized in vitro by Epstein–Barr virus. In total, twenty LCLs of negative controls and the three LCLs of patients carrying *CDH1* VUS have been treated with puromycin, a nonsense-mediated mRNA decay inhibitor. LCL21 and LCL22 have been studied without puromycin treatment. To study *CDH1* VUS c.1936+5G>A, RNA was extracted from formalin-fixed paraffin-embedded (FFPE) tissue blocks of the patient’s breast tumor as we were not able to establish a LCL for the patient. We performed an automated extraction using Maxwell RSC SimplyRNA Blood kit (AS1380) (Promega Corporation, Madison, WI, USA) on Promega Maxwell RSC Instrument for LCLs and Maxwell RSC RNA FFPE kit (AS1440) for FFPE blocks. RNA yield and RNA quality were checked on TapeStation using RNA ScreenTape (Agilent Technologies, Santa Clara, CA, USA). All the samples had DV200 higher than 70% (the percentage of RNA fragments longer than 200bp).

### 2.3. Targeted RNA Sequencing Protocol

#### 2.3.1. RNAseq Library Preparation and Sequencing

RNAseq libraries were prepared on 200 ng of total RNA using SureSelect XT HS2 RNA system kit (Agilent Technologies) according to the manufacturer’s instructions. We used a customized design for the targeted enrichment (SureDesign, Agilent technologies, Santa Clara, CA, USA), capturing exclusively the known exons of 52 cancer predisposition genes, including *CDH1*. No probes targeting spanning exon–exon junctions were used to avoid the bait of enrichment. Sequencing was performed on a NextSeq500 (Illumina Technologies) using Illumina NextSeq mild output kit.

#### 2.3.2. Bioinformatics Pipeline for RNAseq Data Analysis

A Gustave Roussy personalized bioinformatics pipeline (https://github.com/gustaveroussy/DeViSE (accessed on 23 May 2025)) was developed for qualitative and quantitative RNAseq analysis. Fastq data were aligned using STAR v.2.5a on the human genome reference sequence (hg19). A quality filter using Portcullis allowed the selection of good quality splice junctions in a bed file. RegTools software (v0.5.1) was used to annotate the junctions (gene involved, start and end junction genomic positions) and to query gene splicing events reported in Ensembl Genome Browser database (https://grch37.ensembl.org/index.html (accessed on 23 May 2025)). Junctions were then classified into known or unknown splicing junctions. Splicing junctions were visualized using Integrative Genomics Viewer 2.8 (IGV) software.

A quantitative analysis to evaluate the level of expression of splicing events was made following the calculation method reported by Davy et al. [5]. For the description of alternative splicing events of *CDH1* gene, we only reported junctions with at least 100 reads and a ratio of expression higher than 0.1%, similar to the cutoff defined by Davy et al. [5].

To validate our new qualitative and quantitative RNAseq approach, we studied our ability to detect and quantify the alternative splicing patterns of *BRCA1* and *BRCA2* genes. Results were comparable to those reported by Davy et al. [5]. So, we confirmed that our method was sensitive for the detection of alternative splicing patterns and reliable to study their level of expression.

#### 2.3.3. Bioinformatic Validation of the Novel Described Isoforms

We studied the depth and the coverage of the newly described junctions and established the level of confidence for each isoform. All the cited junctions were covered with at least 30 pb on both sides of the junction with a high level of confidence when the depth was higher than 50 reads, and a moderate one when the depth was between 30 reads and 50 reads.

### 2.4. Validation of Highly Expressed CDH1 Alternative Spliced Transcripts

#### 2.4.1. Reverse Transcription PCR (RT-PCR)

Complementary DNA (cDNA) synthesis was performed on 1 µg of total RNA using a transcriptor high fidelity reverse transcriptase and random hexamer primers (Transcriptor High Fidelity cDNA Synthesis kit, ROCHE, Basel, Switzerland) according to the manufacturer’s protocol.

#### 2.4.2. Crystal™ Digital PCR Quantification

We used crystal digital PCR (ddPCR) to quantify the alternative transcript of *CDH1* gene with the skip of exon 11. Two controls already studied by RNAseq were selected (LCL1 and LCL3). Mono-color experiments were performed on a customized Naica Crystal Digital PCR system (Stilla Technologies, Villejuif, France). For each control, we targeted *CDH1* transcripts separately in distinct chambers: transcripts *CDH1* transcript with no exon 11 skipping (probe designed on exon 11), *CDH1* transcript with the skip of exon 11 (probe designed on the junction linking exon 10 to exon 12) and *CDH1* exon 15, a conserved exon used as a reference for the quantification of total *CDH1* transcripts (probe on exon 15) (Table S1). In each hybridization chamber, 3 μL of cDNA was used in 24 μL of PCR mixture combining 1 X PerfeCTa qPCR ToughMix UNG Low ROX (Quanta Biosciences, Gaithersburg, MD, USA) and 0.1 µM of Fluorescein (Sigma, Saint Louis, MO, USA). The final concentration of primer mix for the amplification of the targets was 0.5 µM. Four PCR reactions were loaded per Stilla’s Sapphire chip, compartmentalized in four chambers into 2D monolayers of droplet partitions and thermocycled using the Naica Geode instrument. Cycling conditions were 95 °C for 5 min, followed by 45 cycles of 95 °C for 30 s and 60 °C for 30 s. Sapphire ships were imaged, and data were analyzed as previously described [20].

### 2.5. Characterization of CDH1 Duplication of Exon 4 to Exon 11

#### 2.5.1. RT-PCR and Sanger Sequencing

cDNA was obtained using RNA extracted from the patient’s LCL immortalized in vitro by Epstein–Barr virus and treated with puromycin as described above. Two pairs of primers were designed using Oligo 7 software to target exclusively the abnormal transcript harboring the duplication. The first pair combined a forward primer on exon 11 with a reverse one on exon 4 of *CDH1* gene, and the second pair used a forward primer located on exon 9 and a reverse one on exon 5 (Table S1). PCR amplification was performed using SYBR Green PCR Master Mix (ThermoFisher Scientific, Waltham, MA, USA) and PCR products were analyzed on a High Sensitivity D1000 ScreenTape (TapeStation 2200, Agilent Technologies). Sequencing was performed on an ABI Prism 3730XL automated sequencer (Applied Biosystems, Waltham, MA, USA).

#### 2.5.2. Bionano Optical Genome Mapping

*CDH1* multi-exon duplication was documented using the Bionano optical mapping system (Bionano Genomic, San Diego, CA, USA). Ultra-high-molecular-weight DNA extraction from EBV transformed LCL of the patient, DNA labeling and data collection for optical genome mapping were performed following the manufacturers’ instructions (Bionano Genomics, San Diego, CA, USA). *CDH1* gene was covered with a resolution of 500 pb.

### 2.6. Gene and Alternative Spliced Transcripts Nomenclature

Splicing patterns and variants description were referred to *CDH1* transcript NM_004360.3 (ENST00000261769.5). For the description of alternative splicing events, we used the following nomenclature, as previously described [5,9,10]: (Δx) to refer to the skip of exon “x”; (Δxp) or (Δxq) to describe the loss of the initial or terminal part of exon “x” due to the shift in a splice intronic donor or acceptor site, respectively, and (▼xp) to describe the intronic retention of the initial part of intron “x”.

## 3. Results

### 3.1. Description of Alternative Splicing Patterns of CDH1 Gene

**Figure 1:**
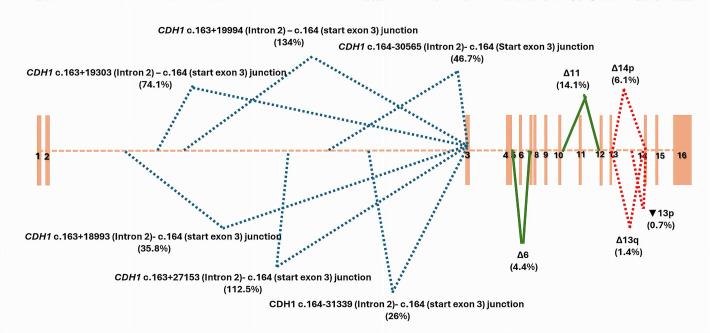
*CDH1* splicing patterns identified by capture RNAseq on 20 negative controls with no pathogenic variant of *CDH1* gene on DNA sequencing. Mean percentage of expression of each alternative splicing event was mentioned between brackets.

In this study, we aimed to describe the alternative splicing patterns of the CDH1 gene. The analysis of *CDH1* junctions’ data of the 20 negative LCL controls treated with puromycin revealed a total of eleven alternative splicing events. The skip of exon 6 (Δ6) and the skip of exon 11 (Δ11) have been found in all the tested LCLs with a mean percentage of expression at 4.4% and 14.1%, respectively (Figure 1, Figure S1). *CDH1* (Δ11) level of expression ranged from 5.8% to 39.5%. Its distribution among the 20 LCLs was detailed in the Figure S1. *CDH1* (Δ6), and (Δ11) splicing events were predicted to cause a truncated protein with a premature stop codon (Table S2). They were found with a high level of confidence using our bioinformatic evaluation. The junctions that defined these exons skipping were expected, as they were specific of known 1 *CDH1* transcripts reported in Ensembl Genome Browser database (https://grch37.ensembl.org/index.html (accessed on 23 May 2025)), *CDH1*-005 (ENST00000566612.1) for *CDH1*(Δ11) and *CDH1*-010 (ENST00000561751.1) for *CDH1*(Δ6). They were reported as nonsense-mediated decay transcripts in the database. *CDH1* (Δ11) was found at low levels on the untreated LCL21 and LCL22 (Table S2), supporting their possible removal by the NMD system.

*CDH1* (Δ14p) alternative splicing event was also described among all the control LCLs. It was found at a low physiological level (6.1%) due to the shift in the consensus splice intronic acceptor site of intron 13, causing the deletion of the first 35 bases of exon 14 (*CDH1* r.2165_2198del). Two alternative splicing events, Δ13q and▼13p, modifying *CDH1* exon 13, have been found among half of the 20 negative control LCLs (Table S2). Δ13q resulted from a shift in the consensus splice exonic donor site of intron 13 creating a terminal truncation of exon 13 with the loss of the last 188 bases (*CDH1* r.1977_2164del). ▼13p created, however, a large insertion into exon 13 due to the shift in the consensus splice intronic donor site of intron 13 and the retention of its 1270 first bases (*CDH1* r.2164_2165ins2164+1_2164+1270). Δ13q and▼13p have been found at very low levels, 1.4% and 0.7%, respectively (Figure 1 and Table S2 ). Δ14p, Δ13q and ▼13p were described with a moderate level of confidence using our bioinformatic evaluation.

**Figure 2:**
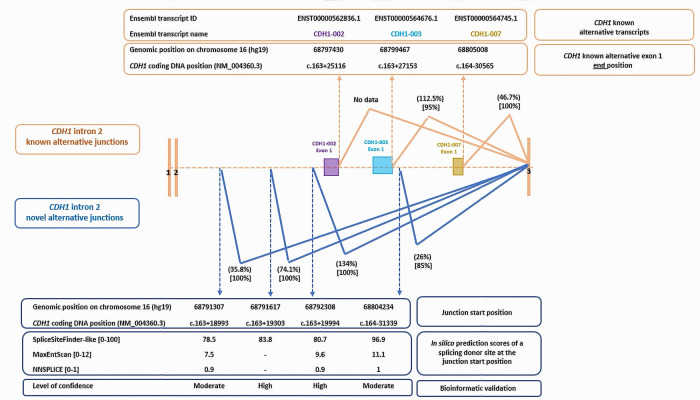
Known and novel alternative splicing junctions within intron 2 of *CDH1* gene: a description of genomic and coding DNA positions, level of expression (between brackets on the top) and recurrence of the splicing event (between brackets at the bottom). In silico prediction scores for splicing donor sites and bioinformatic level of confidence have been detailed for the novel alternative junctions. Known cryptic exons within intron 2 of alternative *CDH1* transcripts were mentioned, and data were extracted from Ensembl Genome Browser database (https://grch37.ensembl.org/index.html (accessed on 23 May 2025)).

We detected six alternative splicing junctions linking a start within *CDH1* intron 2 to the first base of *CDH1* exon 3. Two of them were specific of the exon 1 of reported *CDH1* alternative transcripts (Figure 2). We highlighted four novel alternative junctions within intron 2 that seemed to be involved in the characterization of cryptic exons. As no partner junctions starting from the end of exon 2 have been captured, we supposed that these junctions defined the end of exon 1 of novel isoforms of *CDH1*. In silico scores were highly predictive of a splicing donor site at the start of each junction, strengthening our hypothesis (Figure 2, Figure S2). Interestingly, these junctions have been found among the majority of the LCL controls treated with puromycin, with a recurrence ranging from 85% (17/20 LCLs) to 100% of control LCLs and with a high level of expression varying from 26% to 74,1%, and even exceeding the level of expression of *CDH1* reference transcript for one junction (134% according to the calculation method used in this work) (Figure 2). Three junctions out of the four newly described ones have been found on LCL with no puromycin treatment, and two of them were found with a high level of confidence using our bioinformatic evaluation (Figure 2 and Table S2 ).

### 3.2. Validation and Quantification of the Alternative Splicing Skip of CDH1 Exon 11 (Δ11) Using ddPCR

To confirm and quantify the alternative splicing skip of *CDH1* exon 11 by ddPCR, we selected from the control series two LCL that highly expressed *CDH1* (Δ11) on RNAseq data: LCL1 with a *CDH1* (Δ11) at 39.5% and LCL3 with a *CDH1* (Δ11) at 25%. Mono-color experiments have been performed on total complementary DNA (cDNA) in three separate hybridization chambers following the design detailed in the Figure S3A. We evaluated the proportion of the transcript with a skip of *CDH1* exon 11 (Δ11) to *CDH1* transcript with no exon 11 alternative splicing. Results were close to those quantified by RNAseq (Figure S3B) confirming the physiological high level of expression of *CDH1* (Δ11). In these experiments we also targeted *CDH1* exon 15 (Figure S3A), as this exon was conserved and not involved in a physiological splicing event (Figure 1). Exon 15 was a good candidate for the evaluation of *CDH1* full length transcript. Interestingly, *CDH1* exon 11 skip (Δ11) was present at a level of 40% compared to *CDH1* exon 15 on the tested LCLs, strengthening the conclusion of a high expressed *CDH1* alternative splicing event.

### 3.3. Characterization and Classification of CDH1 Exon 4 to Exon 11 Duplication

We followed a strategy combining RNA analysis and bionano optical genome mapping approach to fully characterize CDH1 exon 4 to exon 11 duplication. This strategy will allow us to determine whether the duplication is in tandem, whether it involves a complex splicing of exon 11, and thus conclude on its clinical pathogenicity. This large rearrangement was not reported in the literature, and therefore was first classified as a variant of uncertain significance.

#### 3.3.1. RNA Analysis Results (RT-PCR and Sanger Sequencing)

The study was made on cDNA using RNA extracted from the patient’s LCL immortalized in vitro with Epstein–Barr virus and treated with puromycin. We first assumed that the duplication would be in tandem, as described for the majority of duplications occurring in breast cancer predisposition genes [21]. Primers were designed to validate this hypothesis combining a forward primer on exon 11 and a reverse primer on exon 5 (Figure S4A.1 and Table S1). After sanger sequencing, we found a junction linking the end of exon 11 to the start of exon 4 on the cDNA chromatogram (Figure S4.B), confirming that *CDH1* exon 4 to exon 11 duplication was direct and in tandem on chromosome 16. A second design of primers was made to study the physiological splicing of *CDH1* exon 11 within the duplication using a forward primer on exon 9 and a reverse primer on exon 5 (Figure S4A.2 and Table S1). The cDNA chromatogram showed a superposition of two distinct transcripts. The first, as expected, was linking exon 11 to exon 4, but the second transcript was harboring a junction linking exon 10 to exon 4, revealing the conservation of the alternative skip of *CDH1* exon 11 within the duplication (Figure S4B.2). A semi-quantitative evaluation of the proportion of the two transcripts showed a level of expression of *CDH1* (Δ11) within the duplication at 28.7% compared to the transcript harboring the duplication and maintaining *CDH1* exon 11 (Figure S2). This was in concordance with the level of expression of physiological *CDH1* (Δ11) described above, on the *CDH1* wild-type LCL treated with puromycin. The two pairs of primers used for this RNA study were specific of the allele harboring the duplication, and no amplification was obtained using a *CDH1* wild-type cDNA control.

Considering these results, *CDH1* exon 4 to exon 11 duplication combined with the physiological splicing of *CDH1* exon 11 within the duplication would generate four possible types of transcripts, depending on the alternative splicing of proximal and distal *CDH1* exons 11 within the duplication (Figure S4C). All the combinations led to a truncated *CDH1* protein (Figure S4C), supporting the conclusion that the duplication of *CDH1* exon 4 to exon 11 has a pathogenic clinical effect.

#### 3.3.2. Bionano Optical Genome Mapping Results

**Figure 3:**
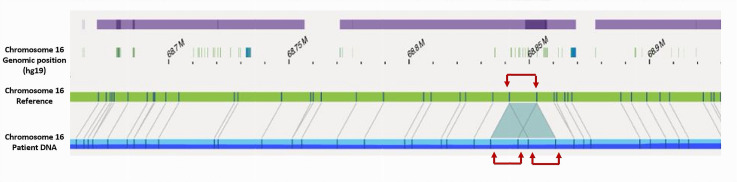
Bionano optical genome mapping alignment of the patient DNA focused on *CDH1* gene against chromosome 16 reference showing in tandem and direct *CDH1* exon 4 to exon 11 duplication (red arrows).

Ultra-high-molecular-weight DNA was extracted from the patient’s LCL immortalized in vitro with Epstein–Barr virus. Bionano optical genome mapping validated RNA results and confirmed that *CDH1* exon 4 to exon 11 duplication was in tandem and direct on chromosome 16 (Figure 3). In addition, this approach allowed a definition of the breakpoint’s positions within *CDH1* intron 3 and intron 11 on genomic DNA (chr16:68841756–68855314). The breakpoints were located within two repeated structures type AluJb (Alu Family; SINE class) suggesting a non-allelic homologous recombination (NAHR) mechanism. In addition, they had deep positions within *CDH1* intron 3 and intron 11, not altering proximal and distal intronic sequences and so probably conserving the major intronic structures of physiological splicing regulation. This may explain the maintenance of the alternative splicing of *CDH1* exon 11 even within the duplication.

#### 3.3.3. RNA Study of CDH1 Splicing Variants of Uncertain Significance

We studied, using RNAseq, the effect on *CDH1* gene splicing of three variants of uncertain significance in patients with an expected effect on *CDH1* gene splicing: *CDH1* c.1008+1G>A; *CDH1* c.1936+5G>A and *CDH1* c.1566-10C>T.

**Figure 4:**
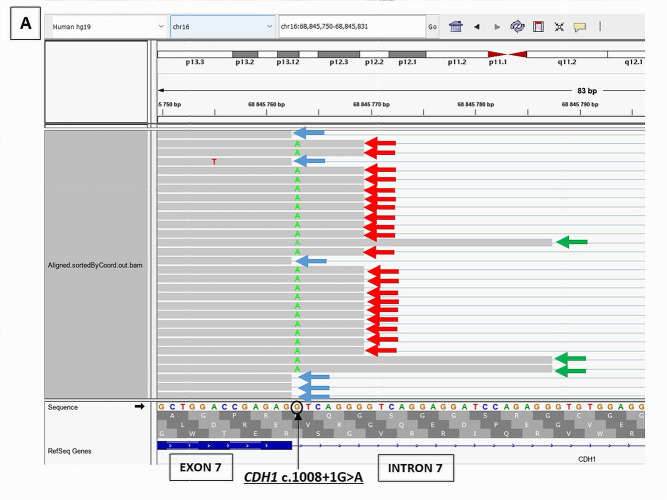

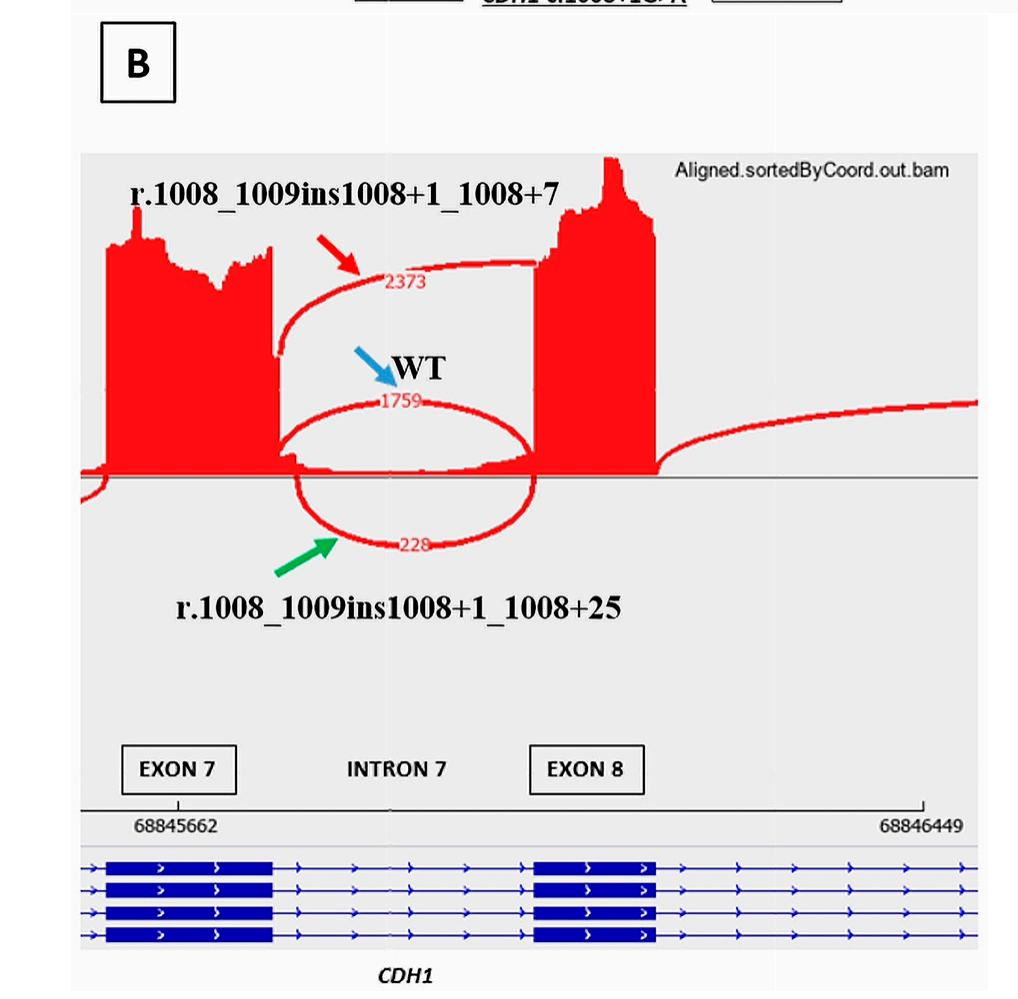
RNAseq results of *CDH1* c.1008+1G>A splicing variant showing the presence of two novel pathogenic transcripts, *CDH1* r.1008_1009ins1008+1_1008+7 (red arrow) and *CDH1* r.1008_1009ins1008+1_1008+25 (green arrow), in addition to the physiological transcript (blue arrow). (**A**): RNAseq BAM alignments in IGV. (**B**): Sashimi Plot showing the splicing junctions between exon 7 and exon 8 of *CDH1* gene and their read coverage.

*CDH1* c.1008+1G>A was predicted by SPiP to cause an alteration of the consensus splice site with a risk of 98.41% [91.47% to 99.96%] for the variant to alter splicing. SpliceAI predicted the loss of the consensus splicing donor site of *CDH1* intron 7 with a significant score of 0.95, suggesting a possible skip of *CDH1* exon 7. However, it has also predicted the gain of a cryptic donor site at *CDH1* DNA codon position c.1008+7, six base pairs upstream from the variant position, with a significant score of 0.33. RNAseq study on a LCL of the patient treated with puromycin, showed the presence of two novel abnormal transcripts starting from the end of exon 7 (Figure 4): *CDH1* r.1008_1009ins1008+1_1008+7 causing the retention of the seven first bases of intron 7 and present at a higher level (2373 reads) than the physiological transcript (1759 reads) and *CDH1* r.1008_1009ins1008+1_1008+25 leading to the retention of the 25 first bases of intron 7 but present at a low level (228 reads). These novel transcripts are considered pathogenic due to their predominance over the physiological transcript and their predicted disruption of the reading frame. RNAseq data did not show a transcript with the skip of exon 7.

**Figure 5:**
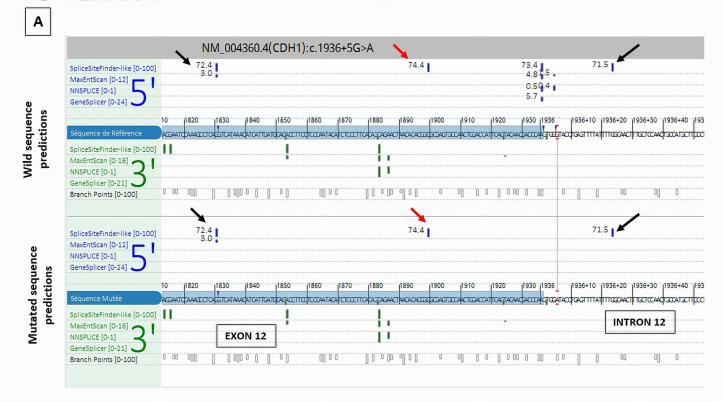

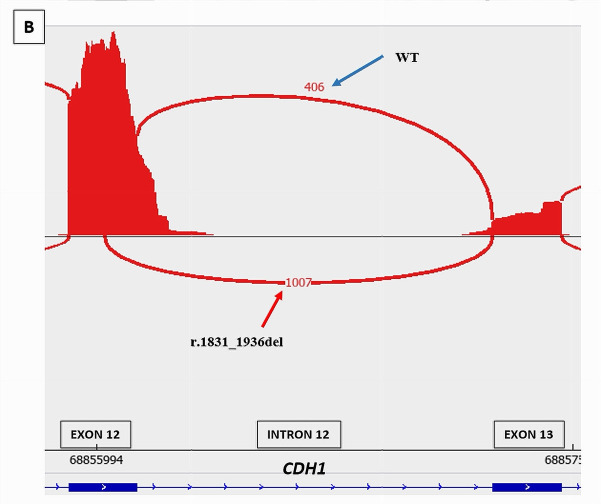
Splicing tools predictions and RNAseq study results of *CDH1* c.1936+5G>A splicing variant. (**A**) Alamut splicing prediction tools showing the loss of the consensus donor splice site of intron 12 and the presence of three cryptic splicing donor sites predicted by SpliceSiteFinder-like tool (read and black arrows). (**B**): RNAseq Sashimi Plot of the splicing junctions between exon 12 and exon 13 of *CDH1* gene and their read coverage showing the presence of a novel pathogenic transcript *CDH1* r.1831_1936del (red arrow) in addition to the physiological transcript (blue arrow).

*CDH1* c.1936+5G>A was predicted by SPiP to lead an alteration of the consensus splice site of *CDH1* exon 12 with a risk of 98.41% [91.47% to 99.96%] for the variant to alter splicing. SpliceAI predicted the loss of the consensus splicing donor site of *CDH1* intron 12 with a significant score of 0.78. This may cause the skip of *CDH1* exon 12. The study of this variant is very interesting as there are three cryptic splicing donor sites predicted by SpliceSiteFinder-like tool on the wild and mutated sequences (Figure 5)—two within exon 12 and one within intron 12—and neither was predicted by SpliceAI to be used in the case of the consensus splicing donor site loss. RNAseq data of the breast tumor revealed the presence of one novel transcript *CDH1* r.1831_1936del due to the use of the cryptic exonic splicing donor site at the DNA codon position *CDH1* c.1830 on exon 12, causing the loss of the last 160 nucleotides of exon 12 (*CDH1* Δ12q). This novel transcript was pathogenic as it was expressed at a higher level (1007 reads) than the physiological one (406 reads) and caused a disruption of the reading frame (Figure 5). We observed a significant imbalance in the expression of *CDH1* pathogenic transcript compared to *CDH1* wild-type transcript, with a high dominance of the pathogenic transcript and a near absence of the normal transcript, suggesting a secondary loss of expression of the wild-type allele, which could be caused by methylation of *CDH1* wild-type allele.

*CDH1* c.1566-10C>T was studied on a LCL of the patient, treated with puromycin as it may affect the spliceosome branch site. No abnormal transcript was found on the RNAseq study. We reviewed the BAM files, and we did not detect any abnormal events involving intron 2.

Regarding RNAseq data, we concluded that *CDH1* c.1008+1G>A and *CDH1* c.1936+5G>A splicing variants are pathogenic. *CDH1* c.1566-10C>T remains of uncertain significance.

## 4. Discussion

We developed a capture RNA-seq approach with a high sensitivity to detect and to quantify physiological and pathogenic splicing junctions in the *CDH1* gene. The results confirmed the presence of main recurrent alternative splice events (Δ11, Δ6) and several known splice variants in the intron 2 region. The ability of this approach to classify uncertain significance variants with an impact on splicing was confirmed in our study by the classification of *CDH1* c.1008+1G>A and *CDH1* c.1936+5G>A as pathogenic splicing variants.

RNAseq results revealed a dynamic and rich splicing profile of *CDH1* large intron 2 of 63,258 pb (Figure 1 and Figure 2). We captured a total of six splicing junctions starting within intron 2 and ending at the start of exon 3. Considering the list of *CDH1* transcripts reported in Ensembl Genome Browser database (https://grch37.ensembl.org/index.html (accessed on 23 May 2025)), in this work, we highlighted four novel splicing junctions within *CDH1* intron 2, with no captured partner junctions starting from the end of exon 2, suggesting that these junctions define the end of exon 1 of novel isoforms of *CDH1*. The high expression levels of these junctions (26%, 35.8%, 74.1% and 134%), along with their notable recurrence rates (observed in 85% to 100% of control LCLs), support the real existence of these splicing events. Furthermore, three of these junctions were detected in LCLs not treated with puromycin, thereby excluding a potential treatment-related bias.

The full transcript cannot be fully described as the splicing events were documented separately using this short-read capture RNAseq approach. Indeed, characterizating exon 1 of novel *CDH1* isoforms requires a third generation RNAseq approach that sequences all the full length isoforms of *CDH1* simultaneously [22,23]. Recently, Aucouturier et al. validated a new approach using a targeted long-read RNA sequencing and listed on their supplementary documents the gross data of *CDH1* full transcripts captured on LCLs [24]. We reanalyzed these raw long-read RNA-seq data and confirmed the presence of the *CDH1* isoforms described in our study, including the novel intron 2-derived isoforms. However, the Δ13q and ▼13p isoforms were not detected. In our own data, they were observed at low expression levels and with moderate confidence based on our bioinformatic analysis, suggesting that they may represent artefactual events. Overall, these preliminary long-read RNA-seq data support the validity of our *CDH1* isoform profiling and reinforce the reliability of our bioinformatic confidence scoring approach. This served as a secondary method of confirmation.

We focused on the skip of *CDH1* exon 11 (Δ11), a major splicing event of *CDH1* gene. *CDH1*(Δ11) was an expected splicing event and was specific to a known *CDH1* transcript, *CDH1*-005 (ENST00000566612.1). In this work, we provided a quantitative evaluation of *CDH1*(Δ11). As it was predicted to induce a truncated *CDH1* protein (*CDH1* p.(Tyr523Phefs*16)), the transcript was reported to be affected by the nonsense-mediated mRNA decay and, therefore, working on puromycin-treated LCLs allowed a better evaluation of the real level of this alternative splicing event. *CDH1*(Δ11) was highly expressed with a mean percentage of 14.1%, reaching from 25% to 39.5%. We confirmed these significant levels using ddPCR, a useful tool for transcripts quantitative evaluation. A tumoral defect of regulation of *CDH1*(Δ11) with a considerable increase in its level of expression has been involved in the tumorigenesis of head and neck cancers [16]. Indeed, physiological splicing events of cancer predisposition genes could be sensitive to splicing dysregulation, producing high levels of aberrant pathogenic transcripts escaping the nonsense-mediated mRNA decay system control [3,25,26].

In our work, we also proposed a new approach combining RNA analysis to Bionano optical genome mapping to classify a large duplication of *CDH1* exon 4 to exon 11. Bionano optical genome mapping is a new technology, which allowed a complete characterization of the large *CDH1* duplication [27] in fast response times, as the analysis was performed on genomic DNA in comparison with RNA analysis that required in vitro immortalization and cell cultures steps. We found, using bionano optical genome mapping, that *CDH1* exon 4 to exon 11 duplication was direct and in tandem on chromosome 16, and we were also able to define the duplication’s breakpoints. However, our comprehensive characterization of *CDH1* alternative splicing isoforms highlighted the potential for complex transcriptional consequences associated with the exon 4 to exon 11 duplication, particularly due to the presence of an alternative exon 11 skipping event. Using an RNA-based approach, we demonstrated that the aberrant allele harboring the *CDH1* exon 4–11 duplication gives rise to four distinct pathogenic transcripts. The study of large multi-exon rearrangements may be challenging, as it may requires a complete knowledge of the gene isoforms in order to remove a possible splicing rescue within the rearrangement that could modify the clinical pathogenicity classification.

*CDH1* gene has a complex alternative splicing profile which varies according to the type of tissue [28,29]. Pinheiro et al. explained that in healthy epithelial tissues, especially in the stomach tissue, the canonical isoform of *CDH1* is mainly expressed with low level of alternative isoforms. On the other hand, they demonstrated the variability of *CDH1* isoforms in non-epithelial tissues by describing alternative isoforms of *CDH1* gene, starting from intron 2 on the spleen tissue, and showed that these isoforms, absent in normal epithelial tissues, could be identified in pathologic conditions and incriminated in the oncogenesis of epithelial tissue tumors when overexpressed [15]. Thus, the four novel isoforms of intron 2 identified in our work on lymphoblastoid cell lines, a non-epithelial tissue, should be investigated to understand their potential impact on tumorigenesis. As *CDH1* intron 2 has been involved in several large rearrangements described in the literature [30] and considering its role in the cis-regulatory mechanisms of E-cadherin functions [15], a complete knowledge of the cryptic exons located within *CDH1* intron 2 in *CDH1* epithelial specific tissues but also in non-epithelial tissues is necessary to fully conclude on the clinical pathogenicity of these reported large rearrangements and deep intronic variants located on these cryptic exons [31].

## 5. Conclusions

Actually, we are witnessing a fast implementation and development of RNAseq approaches in routine laboratories. However, there is an imbalance between the importance of the generated RNA data and the few available reports in the literature describing genes’ isoforms, essential for the interpretation, especially for cancer predisposition genes. We provided in this work a qualitative and quantitative description of alternative splicing patterns of the E-cadherin gene (*CDH1*) on LCLs, using a capture diagnosis RNAseq approach, and highlighted novel isoforms of *CDH1* gene. This description would help to better understand the unclassified variant with a potential splicing impact. The splicing patterns then need to be investigated in tissue for the CDH1 implication in oncogenesis.

## Figures and Tables

**Table 1:** Clinical data and in silico splicing predictions of *CDH1* variants of uncertain significance.

Patient Clinical Presentation	*CDH1* Variants of Uncertain Significance	In Silico Splicing Predictions (SPiP and SpliceAI)
Female patient with a personal history of papillary thyroid cancer (34 years) and bilateral invasive lobular carcinoma (38 years and 40 years)	*CDH1* c.1008+1G>A	SPiP: alteration of the consensus splice site with a risk of 98.41% [91.47% to 99.96%] SpliceAI: loss of the consensus splicing donor site of *CDH1* intron 7 with a significant score of 0.95; and gain of a cryptic donor site at *CDH1* DNA codon position c.1008+7, six base pairs upstream from the variant position with a significant score of 0.33
Female patient with unilateral invasive lobular pleomorphic carcinoma in (39 years) and a metastatic relapse localized at the diaphragmatic cupolas and the ovaries at 51 years	*CDH1* c.1936+5G>A	SPiP: alteration of the consensus splice site of *CDH1* exon 12 with a risk of 98.41% [91.47% to 99.96%] SpliceAI: loss of the consensus splicing donor site of *CDH1* intron 12 with a significant score of 0.78
Female patient with invasive lobular carcinoma at 40 years	*CDH1* c.1566-10C>T	No predictions
Female patient with bilateral invasive lobular carcinoma at 47 years	*CDH1* large duplication from exon 4 to exon 11	Not applicable

## Data Availability

The datasets used and/or analyzed during the current study are available from the corresponding author upon reasonable request, subject to the satisfaction of institutional regulatory requirements.

## Supplementary Materials

The following supporting information can be downloaded at: https://www.mdpi.com/article/10.3390/cancers17203320/s1, Table S1: Sequences of primers and probes used in the study; Figure S1: Distribution of the level of expression of *CDH1* physiological skip of exon 11 (Δ11) among the 20 control LCLs treated with puromycin; Figure S2: Semi-quantitative evaluation of *CDH1* exon 4 to exon 11 duplication transcripts; Figure S3: *CDH1* exon 11 alternative skip (Δ11) validation and confirmation by crystal digital PCR; Figure S4: RNA study of *CDH1* exon 4 to exon 11 duplication; Table S2: *CDH1* alternative splicing junctions’ data identified in the series.

## Abbreviations

LCL	Lymphoblastoid Cell Line
VUS	Variants of Uncertain Significance
NMD	Nonsense-Mediated mRNA Decay
HBOC	Hereditary Breast and Ovarian Cancer
HDGC	Hereditary Diffuse Gastric Cancer syndrome
FFPE	Formalin-Fixed Paraffin-Embedded
