# Supplementary material for: RNA-seq Splicing Profile of the CDH1 Gene and Its Impact on the Clinical Pathogenicity Classification of CDH1 Variants: A Description of Alternative and Pathogenic Splicing Patterns
Source: Cancers (Basel). 2025 Oct 14;17(20):3320. doi: 10.3390/cancers17203320 (PMC12562425; doi:10.3390/cancers17203320)
Supplement: Supplementary file 1 [file cancers-17-03320-s001.zip › cancers-3689697-Supplementary Data_round 2.pdf]

Supplementary Table S1. *CDH1* alternative splicing junctions' data identified in the series.

Genomic position start junction on chr 16 (hg19)	Genomic position end junction on chr16 (hg19)	Junction start (*CDH1* intron/exon, cDNA position)	Junction end (*CDH1* intron/exon position)	Mechanism	Splicing alternative event	RNA predicted alteration	Protein predicted alteration	Recurrence among the 20 control LCLs (puro+)	Mean percentage of the alternative splicing among the LCLs (treated with puromycin) expressing the event	RNAseq statistic data of puro+ LCLs<br>-Mean Junction depth (reads)<br>-Mean Coverage on both sides of the junction (bp)<br>(Alternative / physiological junction)	Percentage of the alternative splicing in LCL21 (No treated with puromycin)	Percentage of the alternative splicing in LCL22 (No treated with puromycin)	Bioinformatic validation: level of confidence	Aucouturier et al, 2024 (long-read RNAseq data)
68791307	68835573	Intron2 (c.163+18993)	Start exon 3	Alternative exon 1 ?	-	r.?	p.?	100 % (20/20)	35,8%	26 reads / 82 reads<br>30 to 50bp / >50bp	0%	0%	Moderate	Detected
68791617	68835573	Intron 2 (c.163+19303)	Start exon 3	Alternative exon 1 ?	-	r.?	p.?	100 % (20/20)	74,1%	64 Reads / 82 reads<br>>50bp / >50bp	4,3%	9,7%	High	Detected
68792308	68835573	Intron 2 (c.163+19994)	Start exon 3	Alternative exon 1 ?	-	r.?	p.?	100 % (20/20)	134%	131 Reads / 82 reads<br>>50bp / >50bp	5,4%	12,5%	High	Detected
68799467	68835573	Intron 2 (c.163+27153)	Start exon 3	Cryptic exon 1 (CDH1-003)	-	r.?	CDH1-003	95% (19/20)	112,5%	89 Reads / 82 reads<br>>50bp / >50bp	0%	16,2%	High	Detected
68804234	68835573	Intron2 (c.164-31339)	Start exon 3	Alternative exon 1 ?	-	r.?	p.?	85% (17/20)	26%	25 reads / 82 reads<br>30 to 50bp / >50bp	0%	0%	Moderate	Detected
68805008	68835573	Intron2 (c.164-30565)	Start exon 3	Cryptic exon 1 (CDH1-007)	-	r.?	CDH1-007	100 % (20/20)	46,7%	35 Reads / 82 reads<br>>50bp / >50bp	0%	0%	High	Detected
68842751	68845587	End exon 5	Start exon 7	Exon skipping	Δ6	r.688_832del	p.(Leu230Glufs*4)	100 % (20/20)	4,4%	75 Reads / 1803 reads<br>>50bp / >50bp	7,46%	3,16%	High	Detected
68849662	68855904	End exon 10	Start exon 12	Exon skipping	Δ11	r.1566_1711del	p.(Tyr523Phefs*16)	100 % (20/20)	14,1%	222 Reads / 1727 reads<br>>50bp / >50bp	7,9%	2,3%	High	Detected
68858799	68862077	Intron 13 (c.2164+1270)	Start exon 14	Splice intronic donor shift	▼13p	r.2164_2165ins2164+1_2164+1270	p.?	65% (13/20)	0,7%	27 reads / 2778 reads<br>30 to 50bp / >50bp	0%	0%	Moderate	Not detected
68857342	68862077	Exon 13 (c.1977)	Start exon 14	Splice exonic donor shift	Δ13q	r.1977_2164del	p.(Val660Phefs*2)	70% (14/20)	1,4%	39 reads / 2778 reads<br>30 to 50bp / >50bp	0%	0%	Moderate	Not detected
68857529	68862111	End exon 13	Exon 14 (c.2199)	Splice intronic acceptor shift	Δ14p	r.2165_2198del	p.(Ile722Argfs*37)	100% (20/20)	6,1%	176 Reads / 2778 reads<br>>50bp / >50bp	1,8%	0,64%	Moderate	Detected

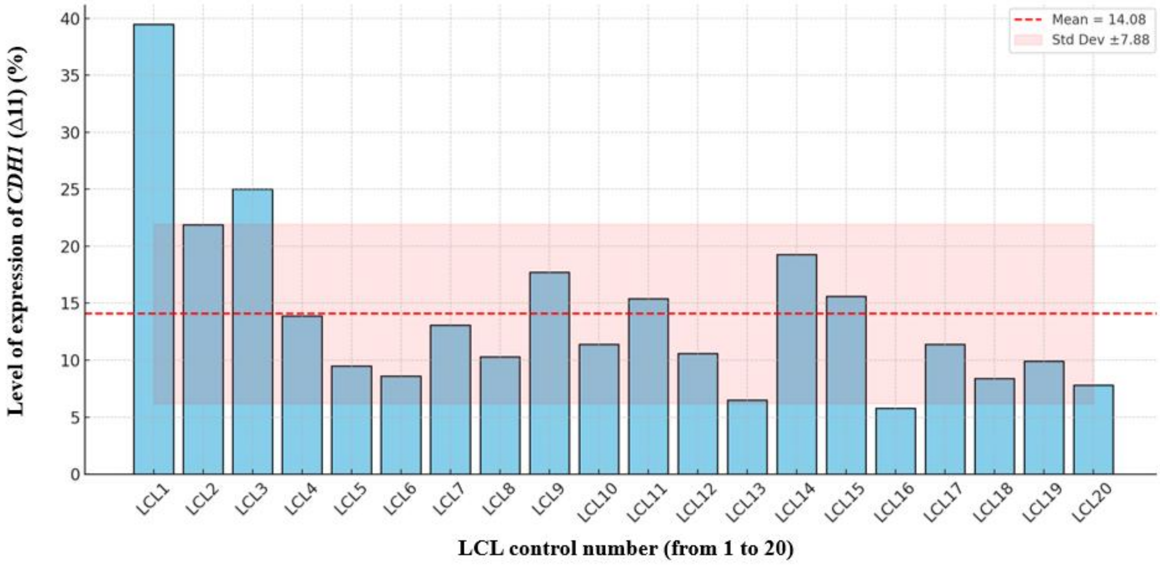

Supplementary Figure S1. Distribution of the level of expression of *CDH1* physiological skip of exon 11 (Δ11) among the 20 control LCL treated with puromycin**.**

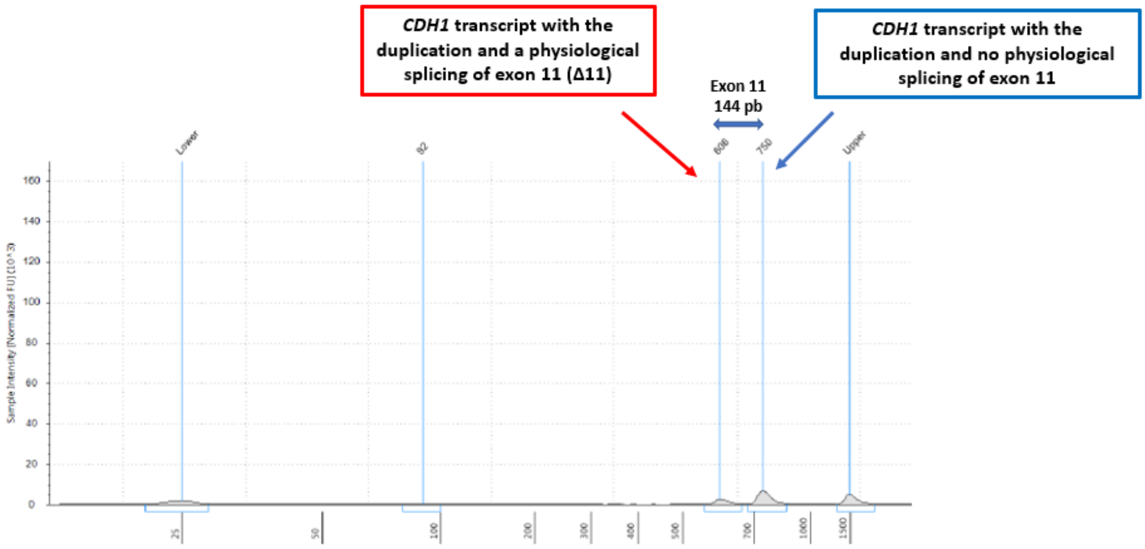

Size [bp]	Calibrated Conc. [ng/µl]	Assigned Conc. [ng/µl]	Peak Molarity [nmol/l]	% Integrated Area	Peak Comment	Observations
25	6.19	-	381	-		Lower Marker
92	0.184	-	3.06	1.34		
606	3.94	-	10.0	28.74		
750	9.59	-	19.7	69.92		
1500	6.50	6.50	6.67	-		Upper Marker

Supplementary Figure S2. Semi-quantitative evaluation of *CDH1* exon 4 to exon 11 duplication transcripts using a High Sensitivity D1000 ScreenTape on TapeStation (Agilent technologies).

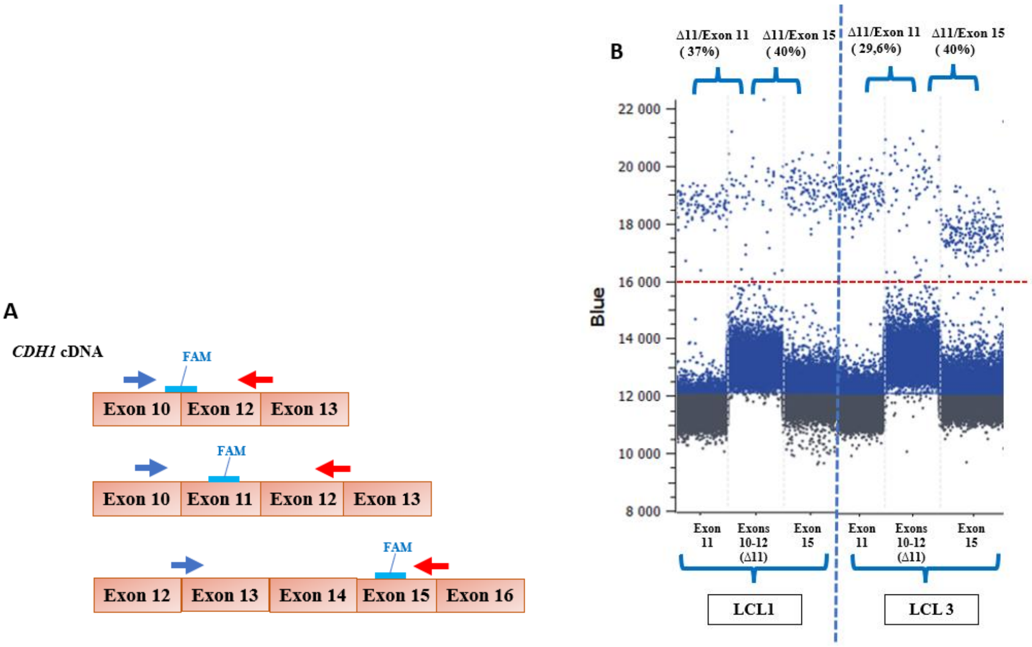

Supplementary Figure S3. *CDH1* exon 11 alternative skip (Δ11) validation and confirmation by crystal digital PCR. A: Location of primers and probes used in each hybridization chamber. Boxes indicate *CDH1* exons, blue arrows indicate the position of forward primers, red arrows indicate the position of reverse primers, blue boxes indicate the position of FAM probes. B: 1D dot plot figure of crystal digital PCR results of the three experiments on LCL1 and LCL3 visualized with CrystalMiner software.

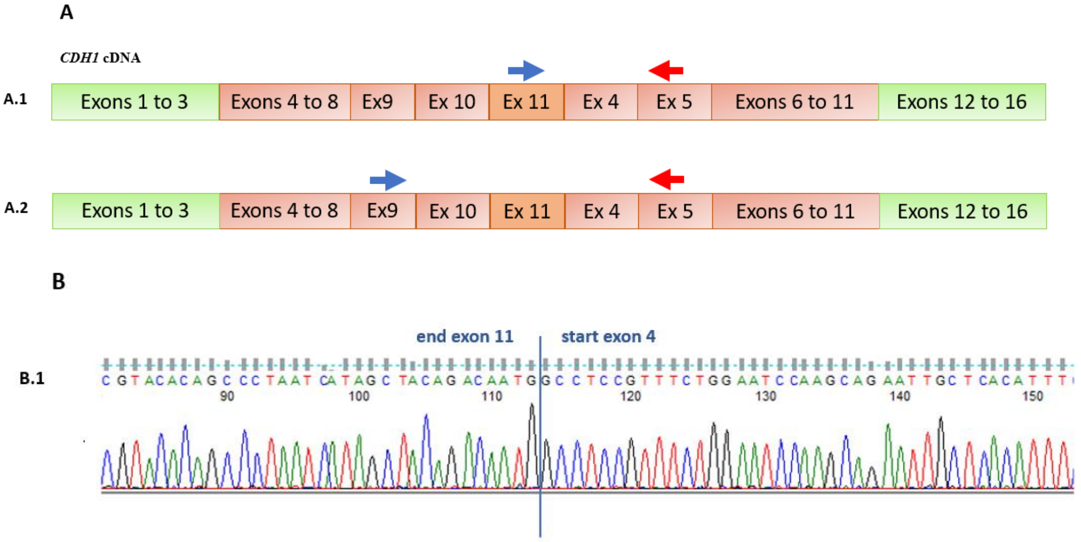

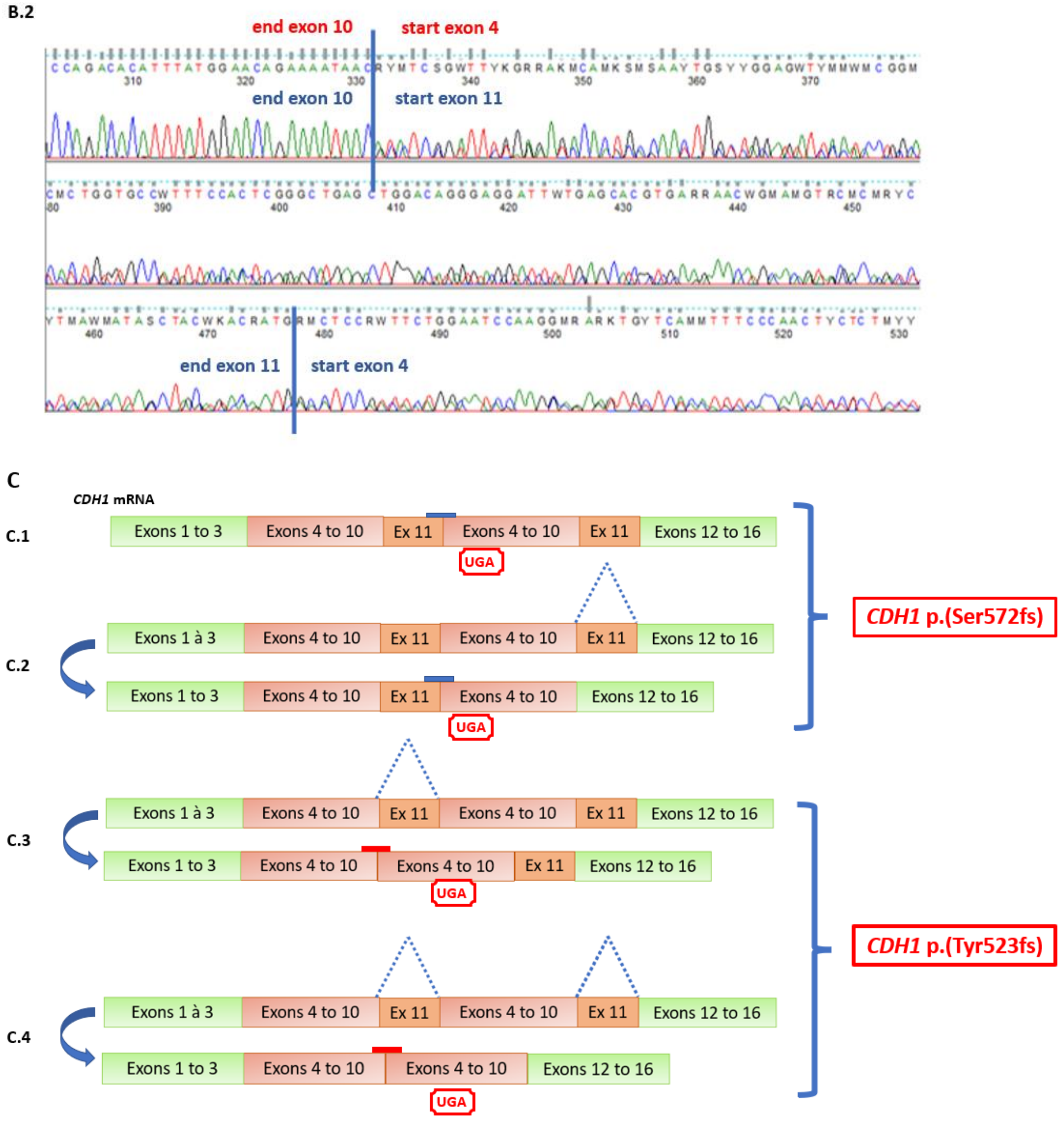

Supplementary Figure S4. RNA study of *CDH1* exon 4 to exon 11 duplication. A. Location of primers used in each experiment. Boxes indicate *CDH1* exons, blue arrows indicate the position of forward primers and red arrows indicate the position of reverse primers. B. cDNA chromatograms showing *CDH1* exon 4 to exon 11 duplication transcripts: a transcript with no alternative splicing of *CDH1* exon 11 (presence of a junction linking exon 11 to exon 4) and a second transcript harboring an alternative splicing of *CDH1* exon 11 (presence of a junction linking exon 10 to exon 4). C.Combination of transcripts resulting from the presence of an alternative splicing of proximal and distal exons 11 within *CDH1* exon 4 to exon 11 duplication. The spliced exon 11 was showed with dotted lines. Blue boxes were located on the junction linking exon 11 to exon 4 and red boxes were located on the junction linking exon 10 to exon 4.
