# Supplementary material for: RNA-seq Splicing Profile of the CDH1 Gene and Its Impact on the Clinical Pathogenicity Classification of CDH1 Variants: A Description of Alternative and Pathogenic Splicing Patterns
Source: Cancers (Basel). 2025 Oct 14;17(20):3320. doi: 10.3390/cancers17203320 (PMC12562425; doi:10.3390/cancers17203320)
Supplement: Supplementary file 1 [file cancers-17-03320-s001.zip › Supplementary Materials_revision file.pdf]

Supplementary Material Table S2. Sequences of primers and probes used in the study.

	**Target on *CDH1* gene**	**Sequence**
**Quantification of the physiological alternative splicing skip of exon 11 of *CDH1* gene using crystal digital PCR**	Forward primer on *CDH1* exon 10	AGCAGTGACGAATGTGGTACCTT
	Reverse primer overlapping *CDH1* exon 12 and exon 13 junction	ATAGATTCTTGGGTTGGGTCGTT
	CDH1 exon 11 probe (FAM)	ATCCGGACACTGGTGCCATTTCC
	Probe overlapping *CDH1* exon 10 and exon 12 junction (FAM)	AACAGAAAATAACGTTCTCCAGTTGCT
	Forward primer overlapping *CDH1* exon 12 and exon 13 junction	AACGACCCAACCCAAGAATCTAT
	Reverse primer overlapping *CDH1* exon 15 and exon 16 junction	ATCAGCCGCTTTCAGATTTTCATCA
	CDH1 exon 15 probe (FAM)	TAACGACGTTGCACCAACCCTCA
**RNA study of *CDH1* exon 4 to exon 11 duplication**	Forward primer on *CDH1* exon 11	CTGCCAACTGGCTGGAGATTAATCC
	Forward primer on *CDH1* exon 9	GTGGGAGGCTGTATACACCATATTG
	Reverse primer on *CDH1* exon 5	TGCTGTAGAAAACCTTGCCTTCTTT
